# The non‐paretic‐hand‐to‐opposite‐ear test: A simple test to detect aphasia and neglect and an indicator of large anterior vessel occlusion in patients with suspected acute stroke

**DOI:** 10.1002/brb3.3450

**Published:** 2024-03-07

**Authors:** Matthias L. Herrmann, Clara Franck, Florian F. Schuchardt, Simone Meier, Max Henningsen, Nicole Wimmesberger, Diana Rau, Hans‐Jörg Busch, Christian A. Taschner, Erik Farin‐Glattacker, Jochen Brich

**Affiliations:** ^1^ Department of Neurology and Clinical Neuroscience, Faculty of Medicine and Medical Center University of Freiburg Freiburg Germany; ^2^ Section of Health Care Research and Rehabilitation Research (SEVERA), Institute of Medical Biometry and Statistics, Faculty of Medicine and Medical Center University of Freiburg Freiburg Germany; ^3^ Department of Emergency Medicine, Faculty of Medicine and Medical Center University of Freiburg Freiburg Germany; ^4^ Department of Neuroradiology, Faculty of Medicine and Medical Center University of Freiburg Freiburg Germany

**Keywords:** aphasia, large vessel occlusion, neglect, paramedic, stroke

## Abstract

**Introduction:**

Aphasia and neglect in combination with hemiparesis are reliable indicators of large anterior vessel occlusion (LAVO). Prehospital identification of these symptoms is generally considered difficult by emergency medical service (EMS) personnel. Therefore, we evaluated the simple non‐paretic‐hand‐to‐opposite‐ear (NPE) test to identify aphasia and neglect with a single test. As the NPE test includes a test for arm paresis, we also evaluated the diagnostic ability of the NPE test to detect LAVO in patients with suspected stroke.

**Methods:**

In this prospective observational study, we performed the NPE test in 1042 patients with suspected acute stroke between May 2021 and May 2022. We analyzed the correlation between the NPE test and the aphasia/neglect items of the National Institutes of Health Stroke Scale. Additionally, the predictive values of the NPE test for LAVO detection were calculated.

**Results:**

The NPE test showed a strong, significant correlation with both aphasia and neglect. A positive NPE test result predicted LAVO with a sensitivity of 0.70, a specificity of 0.88, and an accuracy of 0.85. Logistic regression analysis showed an odds ratio of 16.14 (95% confidence interval 10.82–24.44) for predicting LAVO.

**Conclusion:**

The NPE test is a simple test for the detection of both aphasia and neglect. With its predictive values for LAVO detection being comparable to the results of LAVO scores in the prehospital setting, this simple test might be a promising test for prehospital LAVO detection by EMS personnel. Further prospective prehospital validation is needed.

## Graphical abstract

The non‐paretic‐hand‐to‐opposite‐ear (NPE) test is a simple, rapid and easy‐to‐perform test for the detection of both aphasia and neglect in patients with suspected acute stroke. The results of this study indicate that its predictive values for large anterior vessel occlusion are comparable to the results of prehospital stroke severity scales, making the NPE a promising test for prehospital detection of large anterior vessel occlusion by paramedics.

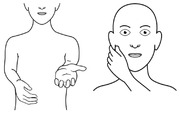

## 1 INTRODUCTION

The cortical symptoms aphasia and neglect combined with hemiparesis are reliable indicators of large anterior vessel occlusion (LAVO) in acute stroke patients (Beume et al., 2018). Prehospital detection of LAVO in patients with suspected acute stroke has an increased priority for emergency medical services (EMS), as the recent American Heart Association/American Stroke Association stroke triage algorithm for EMS recommends the use of a prehospital stroke severity tool to assess for potential LAVO in stroke (Jauch et al., 2021). Although routinely diagnosed by stroke physicians using the National Institutes of Health Stroke Scale (NIHSS) (National Institutes of Health, National Institute of Neurological Disorders & Stroke, 2003) in emergency departments and stroke units, aphasia and neglect are often overlooked in the prehospital setting in acute stroke patients (Dekker et al., 2023; Jia et al., 2017). The examination and correct identification with the NIHSS requirements are perceived by EMS personnel to be difficult and laborious (Birnbaum et al., 2021; Larsen et al., 2022; Purrucker et al., 2017; Wasyliw et al., 2022). Different approaches to testing for aphasia and neglect have been developed as part of various validated LAVO screening scores (Duvekot et al., 2021; Nguyen et al., 2021; Vidale & Agostoni, 2018). However, more than one test is still required for each symptom (e.g., two for aphasia and two for neglect in the Field Assessment Stroke Triage for Emergency Destination [Lima et al., 2016]; the Rapid Arterial Occlusion Evaluation [Pérez de la Ossa et al., 2014]; and the vision, aphasia, and neglect [Teleb et al., 2017] assessment, respectively), making LAVO scores complex and difficult to remember.

To simplify the prehospital identification of aphasia and neglect, we aimed to find one simple, rapid, and reproducible item to test both symptoms, which neither requires severity grading nor specialized neurological expertise. We identified the **n**on‐**p**aretic‐hand‐to‐opposite‐**e**ar (NPE) test as a candidate item because it tests for language comprehension (a subdomain of aphasia) as well as perception/attention in relation to both sides of the body (a subdomain of neglect).

Therefore, the aim of this study was first to evaluate whether the NPE test is associated with the NIHSS items “Best Language” and “Extinction/Inattention”. In addition, as the NPE test includes testing for arm paresis, we evaluated the diagnostic ability of the NPE test to detect LAVO in an unselected cohort of patients with suspected acute stroke.

## 2 METHODS

### 2.1 Study design

This is a prospective, single‐center observational cohort study.

### 2.2 Study setting and patients

The study was conducted at the Emergency Center of the University Hospital of Freiburg, Germany. The University Hospital's Stroke Unit serves as the only stroke unit for the urban district of Freiburg (approximately 350,000 people). In this catchment area, any patient suspected to have a stroke by paramedics is brought directly to our hospital's Emergency Center, regardless of whether there is a suspicion of LAVO or not. Moreover, the hospital serves as a comprehensive stroke center for the area of south‐west Baden‐Württemberg (approximately 1.1 million people). Paramedics throughout the region are trained to use the face arm speech time test (Harbison et al., 2003). This study included consecutive patients aged ≥18 years with suspected acute stroke by paramedics who were brought directly to our Emergency Center between May 2021 and May 2022. Patients transferred from primary stroke centers were excluded from this analysis. Further exclusion criteria were coma and symptom onset more than 24 h before admission. All patients were examined by experienced neurologists immediately after being referred to the emergency center by paramedics.

### 2.3 The non‐paretic hand‐to‐opposite‐ear (NPE) test

**FIGURE 1:**
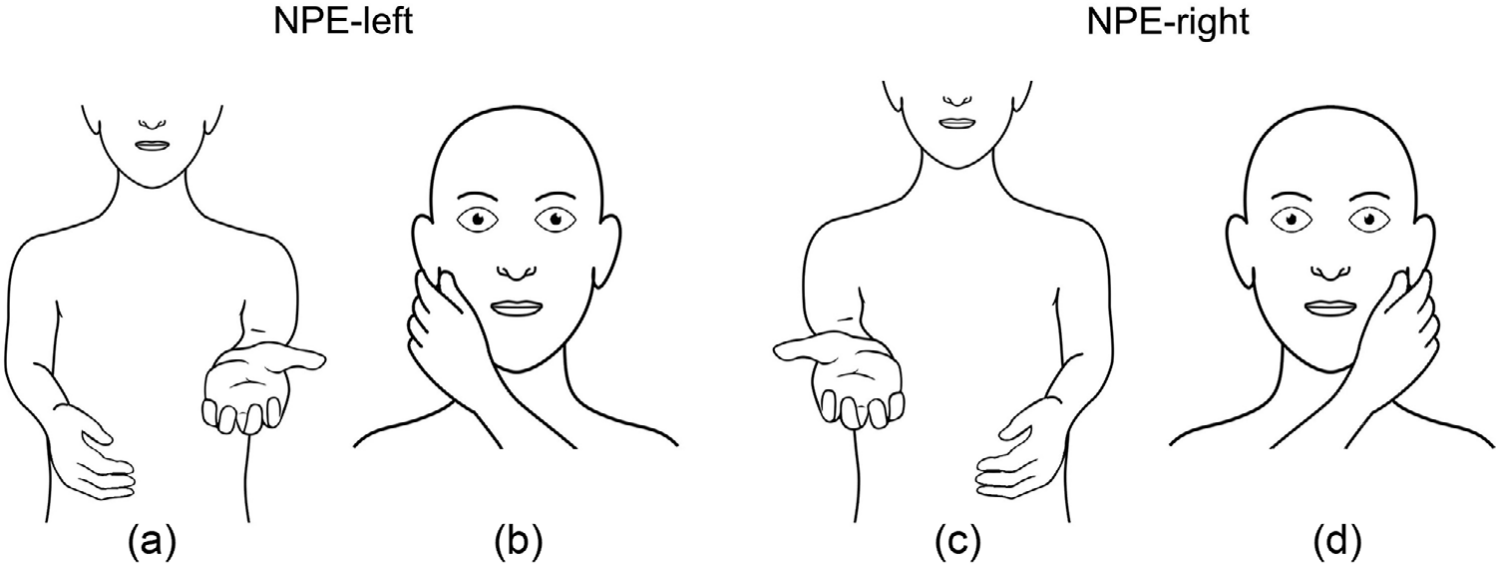
The non‐paretic‐hand‐to‐opposite‐ear (NPE) test. The patient is asked to raise both arms straight and hold them up for 10 s. If the right arm drifts down (a), the patient is immediately instructed to use the left hand to pinch the right earlobe (b, NPE‐left). If the left arm drifts down (c), the patient is instructed to use the right hand to pinch the left earlobe (d, NPE‐right).

The NPE test was derived from a subtest of the test of upper limb apraxia, a standardized 48‐item test used to assess apraxia in clinical practice (Vanbellingen et al., 2010). We modified the test by asking patients to touch the opposite ear instead of the equilateral ear, as originally described. To perform the NPE test, we conducted the pronator drift test to test for arm weakness. In the case of unilateral arm paresis, we immediately instructed the patients to use the hand on the non‐paretic side to pinch the contralateral earlobe (Figure 1). The instruction was repeated once if patients were unable to perform the NPE test correctly on the first attempt. To avoid confusion between left and right, we provided specific instructions regarding the hand and the earlobe that were going to be tested. Therefore, patients with right‐sided paresis were instructed to pinch their right earlobe with their left hand (defined as NPE‐left, Figure 1a,b), whereas patients with left‐sided paresis were instructed to pinch their left earlobe with their right hand (defined as NPE‐right, Figure 1c,d). The NPE test result was considered positive if patients failed to perform the maneuver correctly after the second prompt. Incorrect execution included using the wrong (i.e., paretic) hand, pinching another part of the face (e.g., the nose), pinching the ipsilateral instead of the contralateral earlobe, or not performing the test at all. The test result was considered negative if patients performed the test correctly or if the prerequisite (i.e., unilateral arm paresis of any severity) was not met. We recorded the test result (positive or negative) for each patient.

### 2.4 Neurological examination, imaging, and diagnosis

Each patient was evaluated immediately on admission using both the NPE test and the NIHSS. All patients underwent cerebral imaging by computed tomography (CT) or magnetic resonance imaging, including CT angiography or MR angiography. The images were evaluated by experienced neuroradiologists. We defined LAVO as emergent occlusion of the intracranial carotid artery (ICA), tandem ICA, ICA‐T, or the middle cerebral artery (M1 or proximal M2 segment). The final diagnoses were obtained from the patients’ discharge letters.

### 2.5 Outcomes

To evaluate the association between the NPE test and aphasia/neglect, the NIHSS items “Best Language” and “Extinction/Inattention” served as the primary outcome. The presence of LAVO served as the primary outcome for analyzing the predictive ability of the NPE test.

### 2.6 Statistical analysis

We performed Somers’ delta test in all patients with suspected stroke (Group A, *n* = 1042) to evaluate the association between the NPE and the presence of aphasia (NIHSS item “Best Language”) and the presence of neglect (NIHSS item “Extinction/Inattention”). To evaluate the association between NPE‐left and the presence of aphasia (NIHSS item “Best Language”) and between NPE‐right and the presence of neglect (NIHSS item “Extinction/Inattention”) using Somers’ delta test, we excluded patients with ipsilateral arm paresis. This resulted in a subgroup of 772 patients for the NPE‐left analysis (Group B, consisting of all patients with right arm paresis or without arm paresis), and a subgroup of 808 patients for the NPE‐right analysis (Group C, consisting of all patients with left arm paresis or without paresis). Sensitivity, specificity, accuracy, and positive/negative predictive values (PPV/NPV) were calculated in relation to LAVO for both the NPE test and the comparable symptom combination (arm paresis combined with aphasia and/or neglect) in all patients with suspected stroke (Group A). The symptom combination was assessed using the NIHSS items “Motor Arm” (≥1 point), “Best Language” (≥1 point), and “Extinction/Inattention” (≥1 point). Logistic regression analysis (adjusted for age and sex) was also performed for the NPE test in Group A. To evaluate whether the NPE test allowed identification of both right‐ and left‐sided LAVOs, we analyzed the diagnostic performance of the NPE test and comparable symptom combinations for right‐ and left‐sided LAVOs separately. Left LAVO analysis was performed in Group B (*n* = 772); right LAVO analysis was performed in Group C (*n* = 808). Data were analyzed using the software IBM SPSS Statistics Version 28 (IBM Corporation). Results were considered statistically significant at a level of *p* < .05.

## 3 RESULTS

### 3.1 Patient characteristics

Between May 2021 and May 2022, paramedics brought 1247 patients with suspected stroke to our emergency . We excluded 205 patients for the following reasons: Symptom onset was more than 24 h before admission (*n* = 191); patients were comatose (*n* = 11); clinical data were not available (*n* = 3). Of the 1042 patients included, 468 (44.9%) were female, and the mean age was 73.1 years (standard deviation: 15.2 years). Patients’ diagnoses were distributed as follows: acute ischemic stroke (including transient ischemic attack): *n* = 841 (80.7%); intracranial hemorrhage (ICH): *n* = 83 (8.0%); and stroke mimics: *n* = 118 (11.3%). LAVO was diagnosed in 151 patients (14.5%).

The median NIHSS on admission was 3 (interquartile range [IQR]: 1–9). A total of 504 patients had ≥1 point in the NIHSS item “Motor Arm” with 234 having right arm paresis and 270 having left arm paresis. A total of 272 patients (26.1%) had ≥1 point in the NIHSS‐item “Best Language,” and 260 patients (25.0%) had ≥1 point in the NIHSS‐item “Extinction/Inattention (formerly Neglect).”

A total of 299 patients (28.7%) had the combination of NIHSS items “Motor Arm” ≥1 point (right or left) with “Best Language” ≥1 point and/or with “Extinction/Inattention” ≥1 point. A total of 104 patients (10.0%) had aphasia (“Best Language” ≥1 point) without arm paresis. A total of 24 patients (2.3%) had neglect (“Extinction/Inattention” ≥1 point) without arm paresis. Among patients diagnosed with LAVO, 13 patients (8.6%) had no arm paresis (median NIHSS: 5, IQR: 1.5–10.5).

A total of 214 (20.5%) patients were NPE‐positive (108 NPE‐left and 106 NPE‐right). Table 1 shows the characteristics of patients who tested positive or negative for NPE.

**TABLE 1:** Characteristics of patients with suspected stroke by paramedics who tested positive or negative for non‐paretic‐hand‐to‐opposite‐ear (NPE).

	NPE‐negative (*n* = 828)		NPE‐positive (*n* = 214)
**Unilateral arm paresis**, *n* (%)			
Yes	0 (0)	290 (35.0)	214 (100)
No	538 (65.0)	0 (0)	0 (0)
**Age** in years, mean (± SD)	71.7 (±16.1)	72.3 (±14.5)	77.7 (±13.2)
**Female patients**, *n* (%)	240 (44.6)	123 (42.4)	105 (49.1)
**Diagnosis**, *n* (%)			
Ischemic stroke	431 (80.1)	252 (86.9)	158 (73.8)
Intracranial hemorrhage	28 (5.2)	17 (5.9)	38 (17.8)
Stroke mimics	79 (14.7)	21 (7.2)	18 (8.4)
**LAVO**, *n* (%)	13 (2.4)	32 (11.0)	106 (49.5)
Left‐sided	11 (84.6)	9 (28.1)	57 (53.7)
Right‐sided	2 (15.4)	23 (71.9)	49 (46.3)
**Thrombectomy,** *n* (% of LAVO)			
Left‐sided	4 (36.4)	7 (77.8)	33 (57.9)
Right‐sided	0 (0.0)	17 (73.9)	37 (75.5)
**NIHSS total at admission**, median (IQR)	1 (0–1)	5 (3–8)	16 (12–20)
**NIHSS item “Best Language”,** *n* (%)			
0—no aphasia	432 (80.3)	248 (85.5)	86 (40.2)
1—mild‐to‐moderate aphasia	47 (8.7)	29 (10.0)	10 (4.7)
2—severe aphasia	42 (7.8)	11 (3.8)	41 (19.2)
3—mute, global aphasia	15 (2.8)	1 (0.3)	76 (35.5)
Missing data	2 (0.4)	1 (0.3)	1 (0.5)
**NIHSS item “Extinction/Inattention”,** *n* (%)			
0—no abnormality	511 (95.0)	207 (71.4)	51 (23.8)
1—visual, tactile, auditory, spatial, or personal inattention	19 (3.5)	43 (14.8)	28 (13.1)
2—profound hemi‐inattention or extinction to >1 modality	5 (0.9)	35 (12.1)	130 (60.7)
Missing data	3 (0.6)	5 (1.7)	5 (2.3)

Abbreviations: IQR, interquartile range; LAVO, large anterior vessel occlusion; NIHSS, National Institutes of Health Stroke Scale; SD, standard deviation.

### 3.2 Association between NPE test and cortical symptoms

We found a moderate, significant correlation between a positive NPE test and the presence of ≥1 point for the NIHSS item “Best Language”, or of ≥1 point for the NIHSS item “Extinction/Inattention” (Table 2). When differentiating between NPE‐left and NPE‐right, we found a strong, significant correlation between a positive NPE‐left test and the presence of ≥1 point for the NIHSS item “Best Language” and a positive NPE‐right test and the presence of ≥1 point for the NIHSS item “Extinction/Inattention” (Table 2).

**TABLE 2:** Association between non‐paretic‐hand‐to‐opposite‐ear (NPE) test and aphasia/neglect.

NPE	NIHSS item/cortical symptom	Somers’ d	*p* Value
NPE (all)	“Best Language”/aphasia	.486	< .001
NPE (all)	“Extinction/Inattention”/neglect	.672	< .001
NPE‐left	“Best Language”/aphasia	.854	< .001
NPE‐right	“Extinction/Inattention”/neglect	.751	< .001

Abbreviation: NIHSS, National Institutes of Health Stroke Scale.

### 3.3 Predictive ability of the NPE test for LAVO

Of all 214 patients with a positive NPE test result, 106 (49.5%) were diagnosed with LAVO. A total of 52 patients with ischemic stroke without LAVO were false positive in the NPE test, as well as 38 patients with ICH, and 18 patients were finally diagnosed as stroke mimic. Of the 45 LAVO patients missed with the NPE test, 13 had no arm paresis and matching mild symptoms with a median NIHSS of 5 points (IQR 1.5–10.5). A positive NPE test result identified LAVO overall with a sensitivity of 0.70, a specificity of 0.88, and an accuracy of 0.85 (Table 3). The PPV of the NPE test for LAVO was 0.50, and the NPV was 0.95.

**TABLE 3:** Diagnostic performance of the non‐paretic‐hand‐to‐opposite‐ear (NPE) test for identifying large anterior vessel occlusion (LAVO) compared to the combination of National Institutes of Health Stroke Scale (NIHSS) item “Motor Arm” with cortical symptoms (aphasia and/or neglect).

Group A, *n* = 1042	LAVO (*n* = 151)	No LAVO (*n* = 891)	SEN	SPE	ACC	PPV	NPV
Any arm paresis + aphasia *and/or* neglect	133	165	0.89	0.81	0.83	0.45	0.98
NPE positive	106	108	0.70	0.88	0.85	0.50	0.95

Group B, *n* = 772	LAVO left *n* = 77	No LAVO *n* = 695	SEN	SPE	ACC	PPV	NPV
Right arm paresis + aphasia *and/or* neglect	64	83	0.83	0.88	0.87	0.44	0.98
NPE‐left positive	57	51	0.74	0.93	0.91	0.53	0.97

Group C, *n* = 808	LAVO right *n* = 74	No LAVO *n* = 723	SEN	SPE	ACC	PPV	NPV
Left arm paresis + neglect *and/or* aphasia	69	82	0.93	0.88	0.89	0.46	0.99
NPE‐right positive	49	57	0.66	0.92	0.90	0.46	0.96

*Note*: Group A includes all patients with suspected acute stroke; Group B includes all patients with suspected acute stroke with right arm paresis or without paresis. Group C includes all patients with suspected acute stroke with left arm paresis or without paresis.

Abbreviations: ACC: accuracy; SEN, sensitivity; SPE, specificity; PPV, positive predictive value; NPV, negative predictive value.

Of all 298 patients with the combination of NIHSS item “Motor Arm” ≥1 (right or left) with NIHSS item “Best Language” ≥1 and/or with “Extinction/Inattention” ≥1, 133 (44.6%) were diagnosed with LAVO. A total of 93 patients with ischemic stroke without LAVO were false positive with these combinations, as well as 46 patients with ICH, and 26 patients finally diagnosed as stroke mimic. Of the 18 LAVO patients missed with these symptom combinations, 13 had no arm paresis and matching mild symptoms with a median NIHSS of 5 points (IQR 1.5–10.5). These combinations identified LAVO overall with a sensitivity of 0.89, a specificity of 0.81, and an accuracy of 0.83 (Table 3). When calculated separately, the NPE‐left resulted in sensitivity of 0.74, a specificity of 0.93, and an accuracy of 0.91, whereas the combination of “Motor Arm right” ≥1 with NIHSS‐item “Best Language” ≥1 and/or with “Extinction/Inattention” ≥1 resulted in a sensitivity of 0.83, a specificity of 0.88, and an accuracy of 0.87. The NPE‐right resulted in a sensitivity of 0.66, a specificity of 0.92, and an accuracy of 0.90, whereas the combination “Motor Arm left” ≥1 with “Extinction/Inattention” ≥1 and/or with “Best Language” ≥1 resulted in a sensitivity of 0.93, a specificity of 0.88, and an accuracy of 0.89 (Table 3).

Logistic regression analysis (adjusted for age and sex) revealed a significant association between a positive NPE test outcome and the occurrence of any type of LAVO with an odds ratio of 16.14 (95% confidence interval: 10.82–24.44, *p* < .001).

## 4 DISCUSSION

This study describes a moderate‐to‐strong significant correlation of the NPE test with both NIHSS items “Best Language” and “Extinction/Inattention” and the ability of the NPE test for LAVO prediction in patients with suspected acute stroke.

In the first time‐sensitive contact with acute stroke patients in the emergency department, aphasia is regularly assessed by trained physicians using the NIHSS item “Best Language”. As stated in the original instructions, it is necessary that “the patient is asked to describe what is happening in the attached picture, to name the items on the attached naming sheet and to read from the attached list of sentences. Comprehension is judged from responses here, as well as to all of the commands in the preceding general neurological exam” (National Institutes of Health, National Institute of Neurological Disorders & Stroke, 2003). Analogous to aphasia, neglect is regularly assessed in the emergency setting with the NIHSS item “Extinction/Inattention”. This composite test consists of observing visual spatial neglect or hemi‐inattention to different sensory stimuli, or testing for anosognosia or extinction to bilateral simultaneous stimulation in one of the sensory modalities (National Institutes of Health, National Institute of Neurological Disorders & Stroke, 2003).

Although experienced stroke physicians are able to quickly assess these two NIHSS items as part of the full NIHSS in the emergency setting, acute stroke patients are infrequent events in the daily work of EMS (Zachrison & Goldstein, 2019), resulting in a lack of experience and confidence in clinical assessment. Moreover, the need for observation periods, the provision of additional materials such as pictures or name cards for aphasia testing according to the NIHSS standard, and the need to perform sophisticated examination steps like sensory extinction tests reduce the applicability of these two NIHSS items for the assessment of aphasia and neglect in the prehospital setting.

Validated LAVO screening scores use different approaches to test for aphasia and neglect. However, two or more tasks are typically required to test for aphasia and neglect, respectively (Lima et al., 2016; Pérez de la Ossa et al., 2014; Teleb et al., 2017), making the assessment complex and time‐consuming. Appropriately, it has recently been shown that aphasia and neglect—as assessed with a validated prehospital severity score by EMS—had the weakest correlations with the corresponding NIHSS items “Best Language” and “Extinction/Inattention” assessed by trained physicians in the emergency department (Dekker et al., 2023). To improve this unsatisfactory situation, we propose the NPE test as a simple, dichotomous, short, and easy‐to‐remember test that does not require any additional materials or a long observation period. It addresses different neuropsychological domains because it places a number of demands on the patient. Patients must be able to (1) understand a complex verbal request (language comprehension), (2) plan and execute the movement correctly (praxis), and (3) recognize which side of the body is affected by the arm paresis (whole body/spatial awareness). Conversely, the NPE test covers essential parts for testing the constructs of aphasia, neglect, and apraxia. Because the NIHSS lacks an “apraxia item,” we are unable to specify the additional proportion of apraxia in this study. Nevertheless, from a neuroanatomical point of view, it is clear that testing for language comprehension and neglect, in addition to testing the motor arm areas, covers large areas in both hemispheres (Dronkers et al., 2004; Caggiano & Jehkonen, 2018). This might explain the significant correlations of the NPE test with the multifaceted tests of the NIHSS items “Best Language” and “Extinction/Inattention”.

We believe that our results are particularly interesting from a prehospital perspective because the NPE test fulfills the requirements of a short, dichotomous test that is easy to learn and use without the need for technical support or additional equipment. These characteristics make the NPE test a promising tool for situations where rapid, reliable detection or exclusion of aphasia or neglect is required in the absence of neurological expertise.

Together with the described high sensitivity and specificity for the detection of LAVO by the combination of paresis and cortical signs (Beume et al., 2018), the above characteristics of the NPE test prompted us to test the diagnostic ability of the NPE test for the detection of LAVO in an unselected cohort of patients with suspected acute stroke. Besides replication of the convincing results of Beume et al. (2018) for LAVO detection when combining NIHSS items “Motor Arm” with “Best Language” or “Extinction/Inattention” in our cohort, the much simpler NPE test also demonstrated its ability to detect LAVO. Although the sensitivity is slightly lower than for the combined NIHSS items, the NPE test has high specificity and accuracy. When differentiating between NPE‐right and NPE‐left, no significant difference was found between the detection of LAVO right or LAVO left. Although the sensitivity is rather low for a test performed by neurologists, it is in the upper range of results from other LAVO tests conducted by EMS (Dekker et al., 2023). Because the NPE test is simple to understand and easy to apply without neurological expertise, we speculate that EMS may be able to replicate neurologists’ results to a similar extent. To further improve sensitivity, combining NPE test results with other cortical symptoms such as gaze deviation or additional language tasks (e.g., naming of an object or repeating a sentence) could increase sensitivity without significantly increasing the complexity of the test.

### 4.1 Limitations

There are several limitations. First, the study was conducted in an emergency admission setting of a single comprehensive stroke center, which may limit the generalizability of our results. Second, the NPE test was performed by neurologists rather than emergency medical personnel. We attempted to compensate for this limitation by studying a nonselected cohort of patients, including all patients with suspected acute stroke, which did not differ from that in a prehospital setting. Together with the abovementioned lack of need for neurological expertise, we expect good transferability of the test results. However, a prospective study with EMS personnel in the prehospital setting is needed for external validation. Third, we correlated the NPE test with the NIHSS items “Best Language” for aphasia and “Extinction/Inattention” for neglect, rather than performing comprehensive tests for these symptoms with better symptom characterization. Recently, the NIHSS item “Best Language” was found to be less sensitive than the more comprehensive language screening test (Grönberg et al., 2021), and the NIHSS item “Extinction/Inattention” was found to be less sensitive than the Oxford cognitive screen cancellation task (Moore et al., 2019). However, the NIHSS is the established standard for time‐sensitive assessment of patients with suspected acute stroke in the emergency admission situation, where additional and more complex testing is not feasible. Reassuringly, both publications confirm the ability of the NIHSS items to reliably identify severe forms of aphasia or neglect, which is important for EMS personnel to recognize in the emergency setting and is to be expected in the majority of LAVO stroke patients. This is further underlined by the high sensitivity and specificity of the NIHSS item combinations for the detection of LAVO in our cohort. To gain a better understanding of NPE, as well as to further clarify the apractic component of the NPE test, more detailed tests should be performed simultaneously for each neuropsychological domain in the future. Another limitation of the NPE test is the dependence on unilateral arm paresis for detection of aphasia and neglect, and LAVO. However, aphasia or neglect without arm paresis was rare in our cohort. Overall, 8.6% of LAVO patients had no unilateral arm paresis, leading to false negative results. However, these patients were mostly mildly affected, making the decision for or against thrombectomy a matter of discussion (Hou et al., 2022).

## 5 CONCLUSION

The NPE test is a simple, dichotomous, rapid, and easy‐to‐perform test for the detection of both aphasia and neglect in patients with suspected acute stroke. Our results indicate that this single test predicts LAVO with sufficient sensitivity, and excellent specificity and accuracy. This makes the NPE test a promising approach for prehospital use by EMS personnel. Further prospective prehospital validation is needed.

## AUTHOR CONTRIBUTIONS

**Matthias L. Herrmann**: Conceptualization; data curation; formal analysis; methodology; visualization; writing—original draft; writing—review and editing. **Clara Franck**: Data curation; formal analysis; methodology; writing—review and editing. **Florian F. Schuchardt**: Conceptualization; data curation; writing—review and editing. **Simone Meier**: Conceptualization; data curation; writing—review and editing. **Max Henningsen**: Writing—review and editing. **Nicole Wimmesberger**: Writing—review and editing. **Diana Rau**: Writing—review and editing. **Hans‐Jörg Busch**: Writing—review and editing. **Christian A. Taschner**: Writing—review and editing. **Erik Farin‐Glattacker**: Conceptualization; funding acquisition; methodology; writing—review and editing. **Jochen Brich**: Conceptualization; formal analysis; funding acquisition; supervision; writing—original draft; writing—review and editing.

## CONFLICT OF INTEREST STATEMENT

The authors declare that there are no conflicts of interest.

### PEER REVIEW

The peer review history for this article is available at https://publons.com/publon/10.1002/brb3.3450.

## PATIENT CONSENT

The Ethics Committee waived the need to obtain consent for the present non‐interventional study.

## TRIAL REGISTRATION

The LESTOR trial was registered in the DRKS (German Clinical Trials Register), DRKS‐ID: DRKS00022152.

## Data Availability

The data that support the findings of this study are available from the corresponding author upon reasonable request.

## References

[brb33450-bib-0001] Beume, L.‐A. , Hieber, M. , Kaller, C. P. , Nitschke, K. , Bardutzky, J. , Urbach, H. , Weiller, C. , & Rijntjes, M. (2018). Large vessel occlusion in acute stroke: Cortical symptoms are more sensitive prehospital indicators than motor deficits. Stroke; A Journal of Cerebral Circulation, 49, 2323–2329. 10.1161/STROKEAHA.118.022253 30355088

[brb33450-bib-0002] Birnbaum, L. , Wampler, D. , Shadman, A. , De Leonni Stanonik, M. , Patterson, M. , Kidd, E. , Tovar, J. , Garza, A. , Blanchard, B. , Slesnick, L. , Blanchette, A. , & Miramontes, D. (2021). Paramedic utilization of vision, aphasia, neglect (VAN) stroke severity scale in the prehospital setting predicts emergent large vessel occlusion stroke. Journal of NeuroInterventional Surgery, 13, 505–508. 10.1136/neurintsurg-2020-016054 32611621

[brb33450-bib-0003] Caggiano, P. , & Jehkonen, M. (2018). The ‘neglected’ personal neglect. Neuropsychology Review, 28, 417–435. 10.1007/s11065-018-9394-4 30547412 PMC6327000

[brb33450-bib-0004] Dekker, L. , Daems, J. D. , Duvekot, M. H. C. , Nguyen, T. T. M. , Venema, E. , Van Es, A. C. G. M. , Rozeman, A. D. , Moudrous, W. , Dorresteijn, K. R. I. S. , Hensen, J.‐H. J. , Bosch, J. , Van Zwet, E. W. , De Schryver, E. L. L. M. , Kloos, L. M. H. , De Laat, K. F. , Aerden, L. A. M. , Van Den Wijngaard, I. R. , Dippel, D. W. J. , Kerkhoff, H. , … Zylicz, S. A. (2023). Comparison of prehospital assessment by paramedics and in‐hospital assessment by physicians in suspected stroke patients: Results from 2 prospective cohort studies. Stroke; A Journal of Cerebral Circulation, 54, 2279–2285. 10.1161/STROKEAHA.123.042644 37465998

[brb33450-bib-0005] Dronkers, N. F. , Wilkins, D. P. , Van Valin, R. D. , Redfern, B. B. , & Jaeger, J. J. (2004). Lesion analysis of the brain areas involved in language comprehension. Cognition, 92, 145–177. 10.1016/j.cognition.2003.11.002 15037129

[brb33450-bib-0006] Duvekot, M. H. C. , Venema, E. , Rozeman, A. D. , Moudrous, W. , Vermeij, F. H. , Biekart, M. , Lingsma, H. F. , Maasland, L. , Wijnhoud, A. D. , Mulder, L. J. M. M. , Alblas, K. C. L. , Van Eijkelenburg, R. P. J. , Buijck, B. I. , Bakker, J. , Plaisier, A. S. , Hensen, J.‐H. , Lycklama À Nijeholt, G. J. , Van Doormaal, P. J. , Van Es, A. C. G. M. , … Van Den Biggelaar, J. (2021). Comparison of eight prehospital stroke scales to detect intracranial large‐vessel occlusion in suspected stroke (PRESTO): A prospective observational study. Lancet Neurology, 20(3), 213–221. 10.1016/S1474-4422(20)30439-7 33422191

[brb33450-bib-0007] Grönberg, A. , Henriksson, I. , & Lindgren, A. (2021). Accuracy of NIH stroke scale for diagnosing aphasia. Acta Neurologica Scandinavica, 143, 375–382. 10.1111/ane.13388 33368189 PMC7985870

[brb33450-bib-0008] Harbison, J. , Hossain, O. , Jenkinson, D. , Davis, J. , Louw, S. J. , & Ford, G. A. (2003). Diagnostic accuracy of stroke referrals from primary care, emergency room physicians, and ambulance staff using the face arm speech test. Stroke; A Journal of Cerebral Circulation, 34, 71–76. 10.1161/01.STR.0000044170.46643.5E 12511753

[brb33450-bib-0009] Hou, X. , Feng, X. , Wang, H. , & Li, Q. (2022). Mechanical thrombectomy for mild acute ischemic stroke with large‐vessel occlusion: A systematic review and meta‐analysis. Cerebrovascular Diseases, 51, 615–622. 35378529 10.1159/000523838

[brb33450-bib-0010] Jauch, E. C. , Schwamm, L. H. , Panagos, P. D. , Barbazzeni, J. , Dickson, R. , Dunne, R. , Foley, J. , Fraser, J. F., Lassers, G. , Martin‐Gill, C. , O'Brien, S. , Pinchalk, M. , Prabhakaran, S. , Richards, C. T. , Taillac, P. , Tsai, A. W. , Yallapragada, A. , Prehospital Stroke System of Care Consensus Conference . (2021). Recommendations for regional stroke destination plans in rural, suburban, and urban communities from the prehospital stroke system of care consensus conference: A consensus statement from the American Academy of Neurology, American Heart Association/American Stroke Association, American Society of Neuroradiology, National Association of EMS Physicians, National Association of State EMS Officials, Society of NeuroInterventional Surgery, and Society of Vascular and Interventional Neurology: Endorsed by the Neurocritical Care Society. Stroke; A Journal of Cerebral Circulation, 52, e133–e152. 10.1161/STROKEAHA.120.033228 33691507

[brb33450-bib-0011] Jia, J. , Band, R. , Abboud, M. E. , Pajerowski, W. , Guo, M. , David, G. , Mechem, C. C. , Messé, S. R. , Carr, B. G. , & Mullen, M. T. (2017). Accuracy of emergency medical services dispatcher and crew diagnosis of stroke in clinical practice. Frontiers in Neurology, 8, 466. 10.3389/fneur.2017.00466 28959230 PMC5603652

[brb33450-bib-0012] Larsen, K. , Jæger, H. S. , Hov, M. R. , Thorsen, K. , Solyga, V. , Lund, C. G. , & Bache, K. G. (2022). Streamlining acute stroke care by introducing National Institutes of Health Stroke Scale in the emergency medical services: A prospective cohort study. Stroke; A Journal of Cerebral Circulation, 53, 2050–2057. 10.1161/STROKEAHA.121.036084 PMC9126266 35291821

[brb33450-bib-0013] Lima, F. O. , Silva, G. S. , Furie, K. L. , Frankel, M. R. , Lev, M. H. , Camargo, É. C. S. , Haussen, D. C. , Singhal, A. B. , Koroshetz, W. J. , Smith, W. S. , & Nogueira, R. G. (2016). Field assessment stroke triage for emergency destination. Stroke; A Journal of Cerebral Circulation, 47, 1997–2002. 10.1161/STROKEAHA.116.013301 PMC4961538 27364531

[brb33450-bib-0014] Moore, M. J. , Vancleef, K. , Shalev, N. , Husain, M. , & Demeyere, N. (2019). When neglect is neglected: NIHSS observational measure lacks sensitivity in identifying post‐stroke unilateral neglect. Journal of Neurology, Neurosurgery, and Psychiatry, 90, 1070–1071. 10.1136/jnnp-2018-319668 30674542 PMC6820153

[brb33450-bib-0015] Pérez de la Ossa, N. , Carrera, D. , Gorchs, M. , Querol, M. , Millán, M. , Gomis, M. , Dorado, L. , López‐Cancio, E. , Hernández‐Pérez, M. , Chicharro, V. , Escalada, X. , Jiménez, X. , & Dávalos, A. (2014). Design and validation of a prehospital stroke scale to predict large arterial occlusion. Stroke; A Journal of Cerebral Circulation, 45, 87–91. 10.1161/STROKEAHA.113.003071 24281224

[brb33450-bib-0016] National Institutes of Health, National Institute of Neurological Disorders and Stroke . (2003). NIH stroke scale. National Institute of Neurological Disorders and Stroke. https://www.stroke.nih.gov/documents/NIH_Stroke_Scale_508C.pdf (accessed 8 December 2022)

[brb33450-bib-0017] Nguyen, T. T. M. , Van Den Wijngaard, I. R. , Bosch, J. , Van Belle, E. , Van Zwet, E. W. , Dofferhoff‐Vermeulen, T. , Duijndam, D. , Koster, G. T. , De Schryver, E. L. L. M. , Kloos, L. M. H. , De Laat, K. F. , Aerden, L. A. M. , Zylicz, S. A. , Wermer, M. J. H. , & Kruyt, N. D. (2021). Comparison of prehospital scales for predicting large anterior vessel occlusion in the ambulance setting. JAMA Neurology, 78(2), 157. 10.1001/jamaneurol.2020.4418 33252631 PMC8015863

[brb33450-bib-0018] Purrucker, J. C. , Härtig, F. , Richter, H. , Engelbrecht, A. , Hartmann, J. , Auer, J. , Hametner, C. , Popp, E. , Ringleb, P. A. , Nagel, S. , & Poli, S. (2017). Design and validation of a clinical scale for prehospital stroke recognition, severity grading and prediction of large vessel occlusion: The shortened NIH Stroke Scale for emergency medical services. BMJ Open, 7, e016893. 10.1136/bmjopen-2017-016893 PMC5589005 28864702

[brb33450-bib-0019] Teleb, M. S. , Ver Hage, A. , Carter, J. , Jayaraman, M. V. , & Mctaggart, R. A. (2017). Stroke vision, aphasia, neglect (VAN) assessment—A novel emergent large vessel occlusion screening tool: Pilot study and comparison with current clinical severity indices. Journal of NeuroInterventional Surgery, 9, 122–126. 10.1136/neurintsurg-2015-012131 26891627 PMC5284468

[brb33450-bib-0020] Vanbellingen, T. , Kersten, B. , Van Hemelrijk, B. , Van De Winckel, A. , Bertschi, M. , Müri, R. , De Weerdt, W. , & Bohlhalter, S. (2010). Comprehensive assessment of gesture production: A new test of upper limb apraxia (TULIA): A new test of upper limb apraxia. European Journal of Neurology, 17, 59–66. 10.1111/j.1468-1331.2009.02741.x 19614961

[brb33450-bib-0021] Vidale, S. , & Agostoni, E. (2018). Prehospital stroke scales and large vessel occlusion: A systematic review. Acta Neurologica Scandinavica, 138(1), 24–31. 10.1111/ane.12908 29430622

[brb33450-bib-0022] Wasyliw, S. , Whelan, R. , Davy, K. , Kelly, M. E. , Graham, B. , Gould, L. , & Hunter, G. (2022). The FAST VAN for field identification of large vessel occlusion in acute stroke. Canadian Journal of Neurological Sciences, 50, 1–4. 10.1017/cjn.2022.32 35581931

[brb33450-bib-0023] Zachrison, K. S. , & Goldstein, J. N. (2019). The white whale. Stroke; A Journal of Cerebral Circulation, 50, 1043–1044. 10.1161/STROKEAHA.119.025262 PMC6578575 30917753

